# The Genome Structure of Ciprofloxacin-Resistant Mycoplasma Hominis Clinical Isolates

**DOI:** 10.32607/actanaturae.10941

**Published:** 2020

**Authors:** E. A. Kolesnikova, N. F. Brusnigina, M. A. Makhova, A. E. Alekseeva

**Affiliations:** Academician I.N. Blokhina Nizhny Novgorod Scientific Research Institute of Epidemiology and Microbiology, Federal Service for Surveillance on Customers Rights Protection and Human Wellbeing, Nizhniy Novgorod, 603950 Russia

**Keywords:** Mycoplasma hominis, genome structure, antibiotic resistance mechanisms, gyrA and parC genes, ABC transporters, MATE

## Abstract

The genome structure of three ciprofloxacin-resistant *Mycoplasma hominis *clinical isolates was studied using next-generation sequencing on the Illumina platform. The protein sequences of the studied* Mycoplasma *strains were found to have a high degree of homology. *Mycoplasma hominis *(M45, M57, MH1866) was shown to have limited biosynthetic capabilities, associated with the predominance of the genes encoding the proteins involved in catabolic processes. Multiple single-nucleotide substitutions causing intraspecific polymorphism of *Mycoplasma hominis *were found. The genes encoding the efflux systems – ABC transporters (the ATP-binding cassette superfamily) and proteins of the MATE (multidrug and toxic compound extrusion) family – were identified. The molecular mechanism of ciprofloxacin resistance of the *Mycoplasma hominis *M45 and M57 isolates was found to be associated with the Ser83Leu substitution in DNA gyrase subunit A. In the *Mycoplasma hominis *MH1866 isolate it was related to the Lys144Arg substitution in topoisomerase IV subunit A.

## INTRODUCTION

*Mycoplasma hominis *is one of the most common members of the class *Mollicutes. *Its characteristic features include the absence of a rigid cell wall; an ability to persist on the eukaryotic cell membrane; the small size of their genome; genetic and cell polymorphism; limited metabolic pathways; and antimicrobial resistance (AMR) to drugs that aim to inhibit cell wall biosynthesis [1].

The *Mycoplasma hominis *strains are known to predominantly colonize the urogenital tract in men and women, both healthy ones and those suffering from inflammation (urethritis, cervicitis, vaginitis, bacterial vaginosis, etc.). The ability of *Mycoplasma hominis *to colonize the upper respiratory tract and cause respiratory infections in infants has been proved [1, 2].

Recent data in Russian and foreign publications demonstrate the prevalence of urogenital mycoplasmas resistant to fluoroquinolones and macrolides, the most commonly prescribed antibiotics in the treatment of inflammatory diseases of the pelvic organs [3, 4, 5]. Monitoring antimicrobial resistance in bacteria (including *Mycoplasma hominis*), the pathogens that cause reproductively relevant infections, is a pressing biomedical problem. To resolve it, one needs to study the fundamentals of their pathogenicity, resistance, and adaptation to stressful environments. Modern molecular genetic technologies, next-generation sequencing (NGS) in particular, have made it possible to get closer to understanding these processes. A French research team led by S. Pereyre was the first to sequence and decode the complete nucleotide genome sequence of *Mycoplasma hominis *(*Mycoplasma hominis *ATCC 23114, GenBank accession number FP236530.1) in 2009 [6]. Today, the international GenBank database contains information on complete genome sequences for 23 *Mycoplasma hominis *strains. It should be noted that studying the evolutionary diversity of the *Mycoplasma hominis *population both in Russia and abroad is a challenge, because there are no data on the peculiarities of their genome structure, including pathogenic factors and resistance among the* M. hominis *strains.

The aim of this work was to analyze the genome structure of the ciprofloxacin*-*resistant *M. hominis* clinical isolates found in women with inflammatory diseases of the urogenital tract.

## EXPERIMENTAL

Our study subjects were three *M. hominis *clinical isolates (M45, M57, and MH1866) found in epithelium scraped from the cervical canal of women suffering from inflammatory diseases of the urogenital tract. The women had provided a written informed consent to participate in the study. Commercial differential diagnostic liquid environments manufactured by the Central Scientific Research Institute of Epidemiology, Scientific Research Institute of Epidemiology and Microbiology, Federal Service for Monitoring of Customers Rights Protection and Human Wellbeing (registration number FSR 2008/03366), were used to detect and identify the mycoplasmas, as well as to determine their antibiotic susceptibility pattern. All the studied strains were ciprofloxacin-resistant. The results of a multi-year microbiological monitoring of the prevalence and antibiotic resistance of urogenital mycoplasmas isolated from women and men (both healthy and suffering from inflammatory diseases of the urogenital tract) were reported previously [7-10]. DNA isolation and purification was performed using an AmpliPrime DNA-sorb-V kit (Central Scientific Research Institute of Epidemiology, Federal Service for Monitoring of Customers Rights Protection and Human Wellbeing, Moscow, Russia). Whole genome sequencing was performed on a MiSeq sequencer (Illumina, USA). DNA concentration in the samples was estimated using a Qubit fluorimeter (Invitrogen, Austria). The DNA library for sequencing was prepared using a Nextera XT kit (Illumina, USA). Sequencing was performed using a MiSeq Reagent Kit v2 (Illumina, USA) for 500 cycles. The reference was the whole genome sequence of the *Mycoplasma hominis *ATCC 23114 strain (Gen- Bank accession number FP236530.1). The nucleotide sequences were aligned using the embedded software of the MiSeq sequencer (Isis version 2.6.2.3). Visualization and analysis of the acquired data were performed using the UGENE Unipro [11] and MEGA 7.0 software [12]. The genome annotation was carried out with the help of Rapid Annotation using the Subsystem Technology (RAST) server [13] and the NCBI Prokaryotic Genome Annotation Pipeline (PGAP) (https://www. ncbi.nlm.nih.gov/genome/annotation_prok/). Phylogenetic analysis of the whole-genome nucleotide sequences for the studied strains was conducted using the REALPHY web service [14] Online tool, version 1.12 (https://realphy.unibas.ch/fcgi/realphy). The analysis included all the genome nucleotide sequences of *Mycoplasma hominis *deposited in the RefSeq NCBI database (https://www.ncbi.nlm.nih.gov/refseq). Phylogenetic trees were built with the neighbor-joining algorithm [15] in the MEGA7.0 software [12].

## RESULTS

The whole-genome nucleotide sequences of *M. hominis* have been deposited in the international database NCBI GenBank under the accession numbers MRAY00000000 (*M. hominis *M45), MRAX00000000 (*M. hominis *M57), and QOKO00000000 (*M. hominis* MH1866). The source archives of the reads are available under the numbers SUB 6713744 (M. hominis M57), SUB 6713764 (*M. hominis *M45), and SUB 6713769 (M. hominis MH1866).

Sequencing and assembly of the initial reads made it possible to collect between 18 (MH1866 strain) and 27 (M45 and M57 strains) contigs. It is most likely that the gaps encountered during genome mapping of the* M. hominis *isolates were associated with absence of this region in the original archive of the reads. The size of the genome of the studied strains varied from 633,286 base pairs (M57) to 642,227 base pairs (M45); the GC content was 27.2%. The key metrics of the genome assembly of *M. hominis* are shown in *Table*.

**Table 1:** Structural analysis of the genome of M. hominis clinical isolates (M45, M57, and MH1866)

Characteristic	M. hominis isolates / GenBank accession number			
	MH45/ MRAY00000000	MH57/ MRAX00000000	MH1866/ QOKO00000000	Reference strain ATCC 23114
Assembly length, base pairs	642,227	633,286	639,787	665,445
Number of contigs	27	27	18	1
Coverage	599, 4945	599, 4945	599, 4945	–
Number of reads, million	3.8	3.8	3.8	–
N50	33,392	49,675	57,877	665,445
L50	6	4	4	1
% GC	27.2	27.2	27.2	27.1
Number of genes/pseudogenes	592/28	589/30	581/16	598/12
Number of coding sequences	546	543	546	557
Number of 16S-23S-5S operons	2	2	2	2

An analysis the data acquired using the PGAP (Prokaryotic Genome Annotation Pipeline) server showed that the general number of identified open reading frames was 543 for the *M. hominis *M57 strain and 546 for the *M. hominis *M45 and MH1866 strains, out of which 511 (94.1%), 531 (97.2%), and 534 (97.8%) are accordingly annotated as protein-coding genes. Sixteen pseudogenes were found in the genome structure of the *M. hominis *MH1866 strain; 30 pseudogenes, in the *M. hominis *M57 strain; and 28, in the *M. hominis* M45 strain. Most of the pseudogenes are incomplete nucleotide remnants with unknown functions. However, some of the *M. hominis *pseudogenes were found to contain premature stop codons (three for the *M. hominis* M57 strain) and reading frame shift mutations (four for the *M. hominis *MH1866 strain; two for the* M. hominis *M45 strain; and two for the *M. hominis *M57 strain). Two copies of the 16S-23S-5S rRNA operon were found in the genomes of all strains.

**Fig. 1:**
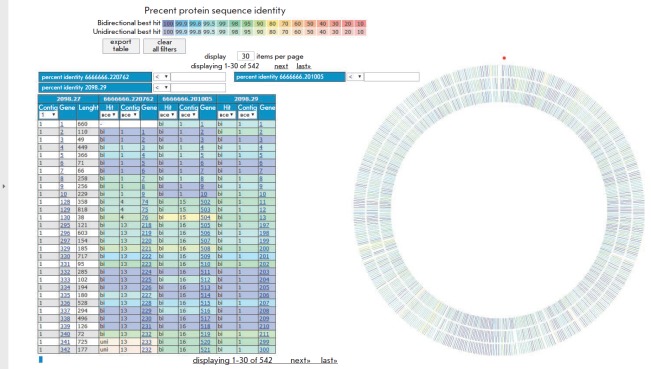
Comparative analysis of the identity of the protein sequences of the *M. hominis *M45, M57, and MH1866 strains. The results were acquired using the RAST server

The genomes of the studied *M. hominis *strains were found to be highly homologous: the degree of protein sequence homology was about 95% (*Fig. 1*).

**Fig. 2:**
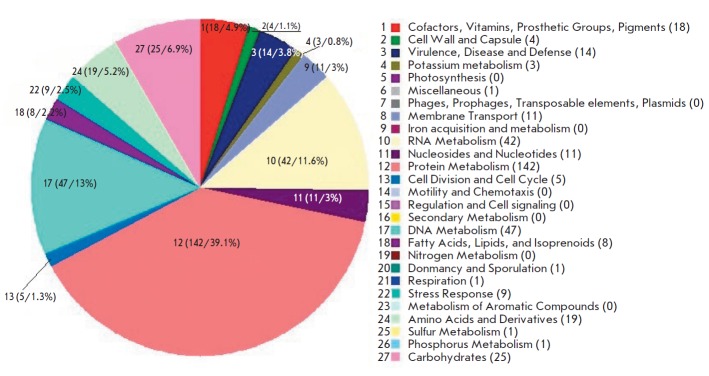
The diagram showing the gene attribution to functional groups for the *M. hominis *MH1866 strain. The picture was acquired using the RAST server. Figures 1–27 are the notation keys for the subsystems in the genome structure. The number of genes in the subsystem / the rate of the genes in the whole genome structure (%) is shown in parentheses

Since the genomes of all three strains have a similar structure, the diagram illustrating gene distribution according to the functions of their product for the* M. hominis *MH1866 isolate is shown in *Fig. 2*.

The functions of the overwhelming majority of the genes in the *M. hominis *genome are related to the synthesis of protein (39.1%), DNA (13%), and RNA (11.6%) (*Fig. 2*). The system of central carbohydrate metabolism of the studied strains (the gene portion is 6.9%) is truncated and consists of individual components involved in the metabolism of pyruvate, pentose phosphates (precursors of ribose and deoxyribose), glucose, and lactose. It was found that the mycoplasma genome contains the *pdP, deoD, deoB, *and *deoC *genes encoding catabolic enzymes: pyrimidine nucleoside phosphorylase, purine nucleoside phosphorylase, phosphopentomutase, and deoxyriboaldolase, respectively. These enzymes participate in the catabolism of deoxyribose and deoxyribonucleoside. The *M. hominis *strains use the 2-deoxy-*D*-ribose portion of the 2’-deoxyribonucleosides resulting from a cascade of biochemical reactions during deoxyribose fermentation as the only source of carbon and energy. The presence of genes encoding enzymes of the purine nucleotide cycle (arginine deaminase (ArcA), ornithine transcarbamylase (ArgF), and carbamate kinase (ArqC)) allows the mycoplasma to derive energy in the form of ATP via an alternative way (arginine and ornithine degradation) [6]. The contribution of the membrane transport products and enzymes involved in the purine metabolism accounts for 3% of the general genome structure.

In every studied mycoplasma isolate, there were genes that encode the efflux systems that participate in membrane transport: namely, ABC transporters (the ATP-binding cassette superfamily) and proteins belonging to MATE (Multidrug and toxic compound extrusion) family). The system of ABC transporters is represented by structural elements performing oligopeptide transport via the bacterial cell membrane: namely, by three copies of the *oppB *gene (encoding the transport proteins of permease OopB) and one copy of the *oppC *gene (encoding the permease OopC). The function of the efflux pumps of the MATE system is ensured through electrochemical gradient of sodions (Na^+^) [16]. The length of the whole sequence of the gene encoding proteins that belong to the MATE family in every analyzed mycoplasma strain is 1,809 nucleotides.

**Fig. 3:**
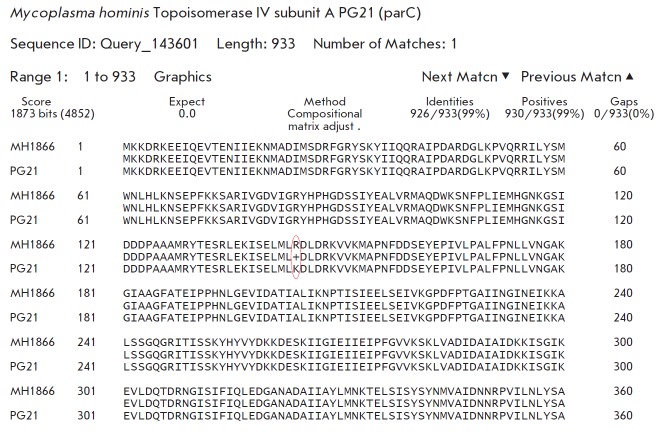
gnment of the amino acid sequence of topoisomerase IV subunit A of the *Mycoplasma hominis* MH1866 clinical isolate and the reference strain* Mycoplasma hominis *ATCC 23114 (PG21). Substitution of lysine (K) for arginine (R) at position 144 is shown in red

As has been stated earlier, the analyzed strains of* M. hominis *(M45 and M57 MH1866) are characterized by resistance to ciprofloxacin. The search for the mutations responsible for resistance to fluoroquinolones was performed by analyzing the QRDR region in the genes encoding topoisomerases: *gyrA *and *gyrB *(DNA gyrase subunits), and *parC *and *parE *(topoisomerase IV subunits). Detailed characteristics of the *gyrA, gyrB, parC *and *parE *genes in the *M. hominis *M45 and M57 isolates were reported in a previously published study [16]. The resistance to ciprofloxacin in the *M. hominis* M45 and M57 isolates was found to be related to the amino acid substitution of serine (S) for leucine (L) at position 83 in DNA gyrase subunit A [16]. It was discovered that the *gyrA, gyrB, parC, *and *parE *genes of the MH1866 isolate contain a great number of nucleotide polymorphisms. Thus, 47 point substitutions were found in the *gyrA *gene; 10 point substitutions, in the* gyrB *gene; 45 substitutions, in the *parC *gene; and 19 substitutions, in the *parE *gene. It was found that the resistance of *M. hominis *MH1866 to ciprofloxacin is attributable to the mutation in the QRDR region of the* parC *gene, which leads to an acid substitution of lysine (K) for arginine (R) at position 144 in topoisomerase IV subunit A (*Fig. 3*).

No meaningful substitutions in the QRDR region of the *gyrA, gyrB, *and *parE *genes in *M. hominis *MH1866 were detected.

**Fig. 4:**
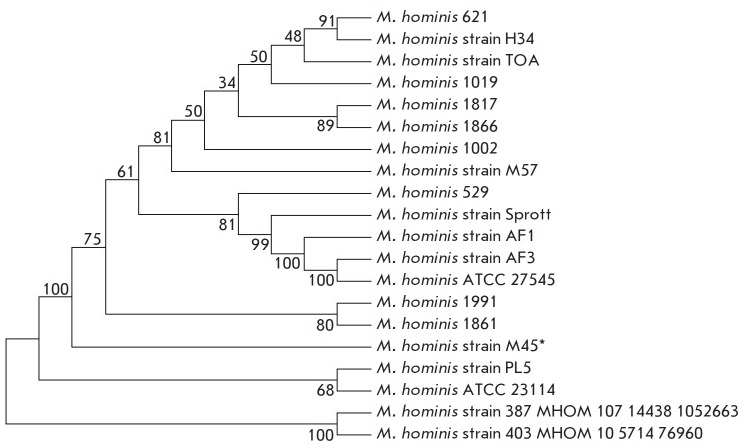
The dendrogram of the whole genome nucleotide sequences for the* Mycoplasma hominis *strains deposited in the international GenBank/NCBI database. * denotes the *Mycoplasma hominis *clinical isolate M45 that is the farthes from other Russian isolates

The dendrogram of the whole genome nucleotide sequences for the studied strains of *M. hominis *(M45 and M57 MH1866) with respect to the *M. hominis *genomes deposited in the GenBank database is presented in *Fig. 4*.

The results of the phylogenetic analysis showed that the *M. hominis *M45 isolate holds a separate position with respect to the Russian mycoplasma isolates and constitutes a separate phylogenetic branch. The* M. hominis *MH1866 isolate is genetically close to the* M. hominis *MH1817 isolate; together, they form a separate cluster. The *M. hominis *M57 strain was singled out into a separate branch within the primary group of Russian mycoplasma isolates.

## DISCUSSION

Modern molecular methods provide an insight into the functioning of the genome of *M. hominis*, one of the smallest prokaryote genomes, and allow researchers to trace its evolution. The French group of scholars led by S. Pereyre was the first to decode the full structure of the genome of *M. hominis *ATCC 23114 (GenBank accession number FP236530.1) in 2009; its size was 665,445 base pairs [6]. As of today (August 23, 2019), the GenBank/NCBI database contains information on the whole genomes of seven *M. hominis *strains and incomplete genomes of 16 strains in the form of contigs (5 strains) and scaffolds (11 strains). The sizes of the genomes of the studied *M. hominis *isolates (M45, M57, MH1866) appear to be smaller than those of the mycoplasma genomes deposited in the Gen- Bank/NCBI. However, the results of a bioinformatic analysis presented in *Table* indicate that the primary characteristics of the genome structure (the number of genes, pseudogenes, RNA, protein coding sequences) of *M. hominis *strains (M45, M57, MH1866) and the reference strain *M. hominis *ATCC 23114 are identical. It should be noted that the data collected in our research agree with the information on other members of the* M. hominis *species represented in the NCBI Genome database (https://www.ncbi.nlm.nih.gov/genome/ genomes/3075?).

The bioinformatic analysis of the genome structure of *M. hominis *(M45, M57, MH1866) has made it possible to uncover a great number of pseudogenes. Pseudogenes are considered to be a reserve of sequences that recombine with functional paralogous genes and thus ensure their genetic diversity [17]. The number of pseudogenes in the genome structure of the *M. hominis* M45 and M57 isolates was found to be twice as large as that in the reference strain. It is possible that numerous mycoplasma pseudogenes ensure assortment of sequences, which is required for creating the genetic diversity of surface antigens [17].

The phylogenetic relationships between the studied strains and the strains whose genomes have been deposited in the GenBank database were evaluated by comparing single-nucleotide polymorphisms. The data of the phylogenetic analysis demonstrate that the studied ciprofloxacin-resistant *Mycoplasma hominis *isolates are genetically heterogeneous. However, a comparative analysis using the RAST server [13] showed a high degree of homology in the protein sequences of the studied mycoplasma strains. A large number of point mutations in the genome of the *Mycoplasma hominis *strains confer them high genetic plasticity and a tendency toward rapid evolution.

The prevalence of genes encoding proteins with catabolic functions in the *Mycoplasma hominis *genome is confirmation that the biochemical capacities of mycoplasmas are scarce. Nutrients are supplied into a mycoplasma cell from the host cells predominantly by transport proteins [1]. The transport proteins of mycoplasma are less specific than the transport proteins of other bacteria; they perform several functions. Thus, the OopB and OopC proteins, which are part of the ABC transporter system, not only implement oligopeptide transportation, but also participate in drug elimination and excretion out of the bacterial cell [18]. When characterizing the non-specific mechanism of fluoroquinolone resistance in *Mycoplasma hominis* (M45, M57, MH1866), one should note that the genome of every studied isolate carries a single copy of the gene encoding multiple drug-resistance proteins MATE. The aforementioned gene is multi-component; it contains homologous sequences of the *Staphylococcus aureus norM *and *mepA *genes, whose role in the excretion of cationic antimicrobial drugs out of the bacterium cell has been proved [19].

It was determined that the molecular mechanism that ensures fluoroquinolone resistance in the studied* Mycoplasma hominis *clinical isolates (M45, M57, MH1866) is possibly related to the nucleotide substitutions in the *gyrA *(S83L) and *parC *(K144R) genes, which changed the amino acid structure of the proteins of the large subunits of DNA gyrase and topoisomerase IV. This mechanism has been identified in and described for a number of conventional bacteria (*E. coli, Streptococcus spp, *and *Staphylococcus spp*.) [20]. The numerous single-nucleotide substitutions in the *gyrA, gyrB, parC*, and *parE *genes of the *Mycoplasma hominis* isolates account for their high degree of genetic polymorphism and play a crucial role in the formation of AMR, which is consistent with the findings reported in publications [21, 22, 23].

## CONCLUSIONS

The results of our research revealed a similarity between the genome structure of the ciprofloxacin-resistant* Mycoplasma hominis *(M45, M57, MH1866) clinical isolates, on the one hand, and the reference strain *M. hominis *ATCC 23114 (GenBank accession number FP236530.1), on the other. We have discovered that genes encoding the proteins involved in catabolic processes are prevalent in the genome structure, which bolsters the aforedescribed theory about the scarcity of biosynthetic capacities in *M. hominis* [1, 6]. In the genome of the studied mycoplasma clinical isolates, we identified a great number of nucleotide substitutions that do not affect amino acid codons, which is indicative of their intraspecies genetic and evolutionary diversity. The studied isolates lack resistance determinants with the conjugative transfer mechanism, which explains the dominance of the conventional molecular mechanism of mycoplasma resistance to fluoroquinolones (ciprofloxacin). This mechanism involves mutations in the QRDR region of the *gyrA *and *parC *genes. It is possible that the identified genes encoding MATE proteins in *M. hominis* can lead to excretion of antimicrobial drugs out of the bacterial cell under certain conditions. It is necessary to conduct such research in order both to understand the natural evolution of *M. hominis *and to gauge the general structure of the urogenital mycoplasma population.

## References

[1] Borchsenius S.N., Chernova O.A., Chernov V.M., Vishnyakov I.E. (2016). Mycoplasmas in biology and medicine at the beginning of the 21st century. St. Petersburg: Nauka, 2016. 333 p..

[2] Belova A.V., Nikonov A.P. (2015). Almanac of clinical medicine..

[3] Zarucheinova OV., Verbov VN., Semenov N.V. (2014). Materials of the scientific-practical conference “From epidemiology to the diagnosis of topical infections ...”..

[4] Baityakov V.V., Syrkina M.G., Radaeva O.A. (2016). Obstetrics. Gynecology..

[5] Lee M.Y., Kim M.H., Lee W., Kang So.Y., Jeon Y.La. (2016). Yonsei Med J..

[6] Pereyre S., Sirand-Pugnet P., Beven L., Charron A., Renaudin H., Barré A., Avenaud P., Jacob D., Couloux A., Barbe V., de Daruvar A., Blanchard A., Bébéar C. (2009). PLoS Genet..

[7] Kolesnikova E.A., Brusnigina N.F. (2014). In the collection: Innovative technologies in anti-epidemic protection of the population. Materials of the All-Russian Scientific and Practical Conference dedicated to the 95th anniversary of the Federal State Budget Scientific Research Institute for Nuclear Power Engineering named after Academician I.N. Blokhina FBUN “Nizhny Novgorod Research Institute of Epidemiology and Microbiology named after Academician I.N. Blokhina”..

[8] Kolesnikova E.A., Brusnigina N.F., Efimov E.I. (2016). In the collection: Modern technologies in epidemiological supervision of topical infections Materials of the All-Russian scientific and practical conference dedicated to the 95th anniversary of the birth of academician RAMN I.N. Blochina. Editorial Board: E.I. Efimov, G.I. Grigor’yeva, N.N. Glukhov, Ye.N. Filatova, V.V. Koroleva..

[9] Kolesnikova E.A., Brusnigina N.F., Efimov E.I. (2018). Russian medical journal. Medical Review..

[10] Kolesnikova E.A., Brusnigina N.F., Kishoyan K.G. (2019). In the collection: Scientific support of the anti-epidemic protection of the population: current problems and solutions The collection of scientific papers of the All-Russian Scientific and Practical Conference with international participation dedicated to the 100th anniversary of the Federal State Budget Scientific Research Institute for Nuclear Power Engineering named after Academician I.N. Blokhina Rospotrebnadzor..

[11] Okonechnikov K., Golosova O., Fursov M. (2012). J. Bioinformatics..

[12] Kumar S., Stecher G., Tamura K. (2016). Mol. Biol. Evol.10.1093/molbev/msw054.

[13] Aziz R.K., Bartels D., Best A.A., DeJongh M., Disz T., Edwards R.A., Formsma K., Gerdes S., Glass E.M., Kubal M. (2008). BMC Genomics..

[14] Bertels F., Silander O.K., Pachkov M.l., Rainey P.B., Nimwegen E. (2014). Mol. Biol. Evol..

[15] Saitou N., Nei M. (1987). Mol. Biol. Evol..

[16] Kolesnikova E.A., Brusnigina N.F., Makhova M.A., Alekseeva A.E. (2018). Clinical microbiology and antimicrobial chemotherapy..

[17] Balakirev E.S., Ayala F.J. (2004). Pseudogenes: conservation of structure, expression and function.. Journal of General Biology..

[18] Raherison S., Gonzalez P., Renaudin H., Charron A., Bébéar C., Bébéar C.M. (2005). Antimicrobial Agents and Chemotherapy..

[19] Sun J., Deng Z., Yan A. (2014). Biochemical and Biophysical Research Communications..

[20] Yoshida H., Bogaki M., Nakamura M. (1990). Antimicrobial Agents and Chemotherapy..

[21] Meng D.Y., Sun C.J., Yu J.B., Ma J., Xue W.C. (2014). Brazilian Journal of Microbiology..

[22] Chernova O.A., Medvedeva E.S., Mouzykantov A.A., Baranova N.B., Chernov V.M. (2016). Acta Naturae..

[23] Rakhmatulina M.R., Kirichenko S.V. (2013). Bulletin of Dermatology and Venereology..

